# Ce^3+^/Yb^3+^/Er^3+^ triply doped bismuth borosilicate glass: a potential fiber material for broadband near-infrared fiber amplifiers

**DOI:** 10.1038/srep33865

**Published:** 2016-09-20

**Authors:** Yushi Chu, Jing Ren, Jianzhong Zhang, Gangding Peng, Jun Yang, Pengfei Wang, Libo Yuan

**Affiliations:** 1 Key Lab of In-fiber Integrated Optics, Ministry Education of China, Harbin Engineering University, Harbin 150001, China; 2 Photonics & Optical Communications, School of Electrical Engineering & Telecommunications, University of New South Wales, Sydney 2052, NSW, Australia

## Abstract

Erbium doped bismuth borosilicate (BBS) glasses, possessing the broadest 1.55 μm near infrared (NIR) emission band among oxide glasses, stand out as excellent fiber material for optical fiber amplifiers. In this work, we demonstrate that both broadened and enhanced NIR emission of Er^3+^ can be obtained by sensibly combining the effects such as mixed glass former effect, phonon-assisted energy transfer (PAET) and de-excitation effect induced by codopant. Specially, by codoping CeO_2_ in a controlled manner, it leads to not only much improved optical quality of the glasses, enhanced NIR emission, but also significantly suppressed energy transfer up-conversion (ETU) luminescence which is detrimental to the NIR emission. Cerium incorporated in the glasses exists overwhelmingly as the trivalent oxidation state Ce^3+^ and its effects on the luminescence properties of Er^3+^ are discussed. Judd-Ofelt analysis is used to evaluate gain amplification of the glasses. The result indicates that Ce^3+^/Yb^3+^/Er^3+^ triply doped BBS glasses are promising candidate for erbium doped fiber amplifiers. The strategy described here can be readily extended to other rare-earth ions (REs) to improve the performance of REs doped fiber lasers and amplifiers.

Since Payne *et al*. first demonstrated the feasibility of erbium doped fiber amplifiers (EDFAs) working at 1.55 μm near-infrared (NIR) wavelength^1^, a wide range of applications in telecommunications, medical surgery, eye-safe measurement and spectroscopy have been realized^2^,^3^. Nowadays, research is mainly oriented in two directions: either broaden the gain spectrum or enhance the output power of Er^3+^ doped fibers, to meet the insatiable demand for optical data transmission capacity and high power lasers. However, the gain width of conventional SiO_2_-based EDFAs is restricted to 40 nm^4^, limiting the wavelengths that can be simultaneously amplified^5^. In contrast, Er^3+^ doped fluoride, tellurite and especially bismuth glasses exhibit gain spectra wider than 50, 70 and 80 nm, respectively^6^,^7^,^8^, thus are more promising candidates for broadband EDFAs.

When it comes to lasing efficiency, although codoping with Yb^3+^ has been routinely applied^9^, the quasi-three-level scheme of Er^3+^ still suffers from the problems limiting the NIR lasing efficiency, such as the population inversion bottle neck between the 1.55 μm lasing level ^4^I_13/2_ and the ground level ^4^I_15/2_, the undesirable energy transfer up-conversion (ETU) emission and excited state absorption (ESA). Recent studies have focused on methods speeding up the non-radiative transition (multi-phonon relaxation, MPL) between the 980 nm pumping level ^4^I_11/2_ and the lasing level ^4^I_13/2_ to increase the population of the latter. Since the multi-phonon relaxation rate is exponentially proportional to the maximum phonon energy of host materials^10^, utilizing comparatively high phonon energy (HPE) materials is a sensible choice, for example, conventional SiO_2_-based EDFAs have representative vibrational phonon energy 1100 cm^−1^, higher than that of tellurite (700 cm^−1^) and fluoride (500 cm^−1^) fibers, it can provide gain amplification up to more than 1000 times easily. Borate crystals of even higher phonon energy (~1350 cm^−1^), such as Na_2_B_4_O_7_^11^, Ca_4_YO(BO_3_)_3_ (YCOB) and YAl_3_(BO_3_)_4_ (YAB), have been demonstrated as excellent laser materials too^12^,^13^. The HPE also brings in an added merit that it effectively restrains the deleterious ETU emission and/or ESA.

Another way to increase the population of the lasing level ^4^I_13/2_ is by addition of a suitable codopant, such as Ce^3+^, Eu^3+^ or Tb^3+^, to accelerate the non-radiative decay ^4^I_11/2_ → ^4^I_13/2_ via phonon-assisted energy transfer (PAET). It has been found that all of the three codopants can decrease the lifetime of ^4^I_11/2_ level, however, only does Ce^3+^ hardly influence the lifetime of the lasing level ^4^I_13/2_, thus standing out as the optimal choice^14^. Codoping of Ce^3+^ has been widely applied to improve the NIR lasing efficiencies of optical fibers^15^, glasses^14^,^16^, glass-ceramics^17^ and crystals^18^. It also helps to suppress the ETU emission and ESA, for example, the very strong ETU emission from Er^3+^ doped NaGd(WO_4_)_2_ crystal almost disappeared completely, and the lasing properties were strongly improved upon the codoping of Ce^3+^ 19.

Having briefly reviewed the recent studies on attaining the broadened and enhanced NIR emission of Er^3+^ in various hosts, we chose bismuth borosilicate (Bi_2_O_3_-B_2_O_3_-SiO_2_, BBS) glass system as potential fiber material for EDFAs. This system has some attractive properties, such as raw materials being relatively cheap, large refractive index (>2.0), excellent fiber drawing ability, being fusion splicable to silica-based fibers and intrinsically photosensitive that entails direct writing of fiber Bragg gratings in the same gain media. Besides, since the system contains HPE compounds B_2_O_3_ and SiO_2_, it is expected that the average phonon energy of the glasses will be large.

Cerium oxide (CeO_2_) was added into the system, serving as an oxidant to against darkening/crystallization of BBS glasses which limits to a great extent the optical applications of these glasses^20^, but also as the ion source for Ce^3+^. The latter point deserves some comments, as is known that cerium incorporated into the glass network is possibly present in two oxidation states, Ce^3+^ and Ce^4+^, and their ionic equilibrium depends on the condition of glass formation and the type of glass system involved^21^. However, we will show in this work that cerium presented overwhelmingly in trivalent oxidation state Ce^3+^ in the BBS glasses. Our results also demonstrate that by resorting to the mixed glass former effect (inhomogeneous broadening) a very broad NIR emission can be obtained in the designed glasses, and when CeO_2_ was doped in a controlled manner both enhanced NIR and dramatically weakened ETU emissions can be observed. Based on Judd-Ofelt (JO) analysis, it is concluded that the lasing properties of the glasses can be improved upon the codoping of CeO_2_.

## Experiments

Glass samples of (40-x)Bi_2_O_3_-50B_2_O_3_-10SiO_2_-xCeO_2_-1Yb_2_O_3_-0.5Er_2_O_3_ (x = 0, 0.1, 1, 3, 5, 10, 15, 20 in mol.%) were prepared by conventional melt-quenching method. The raw materials were weighed 20 g, mixed in an agate mortar for at least 15 min., stored in an alumina crucible and then put in an electric furnace. To prevent evaporation of Bi_2_O_3_, the materials were pre-sintered at 450 °C for 30 min., samples with varying content of CeO_2_ were melted at different temperatures in a range from 950 to 1550 °C. The reason to increase the melting temperature is to dissolve more concentration of CeO_2_, otherwise the samples suffer from serious crystallization (see Results part below). All the obtained glasses were annealed around T_g_ for 180 min. Finally, the glasses were cut and polished carefully to meet the requirements for optical measurements.

Absorption spectra were measured by a Perkin-Elmer Lambda 950 UV-VIS spectrophotometer over the spectral range of 300–2000 nm. Emission spectra were measured by an Edinburgh FS920 fluorescence spectrometer equipped with a liquid-nitrogen cooled steady state InSb detector and luminescence decay was recorded by a TDS3000C digital phosphor oscilloscope. Sample density was obtained by Archimedes method with alcohol as the immersion liquid. Er^3+^ concentration was calculated from the measured density and initial composition. The X-ray photoelectron spectra spectroscopic (XPS) was measured by ESCALAB250Xi (Thermo Fisher U.S.), the glass samples were broken in the XPS chamber and only those newly created cross-sections were measured. Raman spectra were measured by RenishawInvia Raman microscope (Renishaw, Gloucestershire, UK) with the excitation wavelength of 515 nm. All the measurements were carried out at the room temperature.

## Results

**Figure 1:**
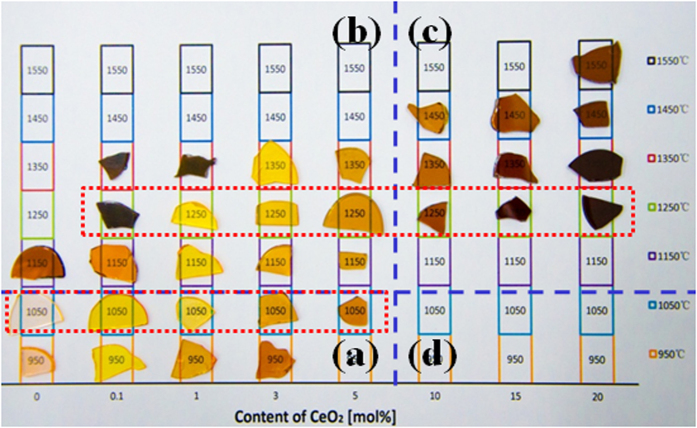
Digital photo of the obtained glasses doped with different concentration of CeO_2_ (increasing from the left to the right) and melted at different temperatures (increasing from the bottom to the top). The empty space implies that it is not possible to obtain the glasses with the indicated compositions and MT due to severe devitrification (crystallization).

Figure 1 shows coloration of the glasses with varying content of CeO_2_ and melted at different temperatures. Depending on the content of CeO_2_ and melting temperature (MT), the figure can be divided into four parts. At high MT (>1200 °C) and low CeO_2_ content (<5 mol.%) region (b), the glasses suffer from serious darkening/devitrification and cannot be obtained due to the evaporation of Bi_2_O_3_; at high MT and CeO_2_ content region (c), the addition of CeO_2_ markedly improves the transparency and avoids the devitrification of the glasses. Obviously, the solubility of CeO_2_ increases with MT such that up to 20 mol.% CeO_2_ can be incorporated into the glasses. At low MT and CeO_2_ content region (a), on the other hand, the addition of CeO_2_ increases the absorption of the glasses in the visible region. Finally, glasses cannot be prepared at low MT and with high CeO_2_ content (d) due to severe crystallization resulted from the undissolved CeO_2_.

The XRD patterns of the samples sitting on the border separating those that can form glass and those cannot were measured (cf. Fig. S1). The amorphous nature of the glasses was confirmed. DSC curves of some representative samples (showing the best NIR emission properties) were also measured (cf. Fig. S2 and Table S1). The ΔT = T_x_ − T_g_ (T_x_, onset temperature of crystallization and T_g_, glass transition temperature) values were considerably larger than 100 °C, indicating excellent thermal stability and fiber drawing potential. On the other hand, as is known, traditional bismuth glasses have relatively low melting (<1000 °C) and glass transition temperatures (<450 °C), the glasses volatilize and the optical properties worsen terribly when melted or handled at high temperatures, all of which pose a serious barrier to fiber drawing and fusion with conventional silicate optical fibers^8^. As shown in Fig. 1, addition of a moderate amount of CeO_2_ (e.g., 3 mol.%) makes excellent glasses even when melted at temperature as high as 1350 °C. And the glass transition temperature can be significantly increased up to 638 °C for the glass containing CeO_2_ up to 20 mol.% (Table S1). Therefore, the advantage to increase the melting and glass transition temperatures is to mitigate the difficult in the fiber drawing and fusion with conventional silicate fibers.

**Figure 2:**
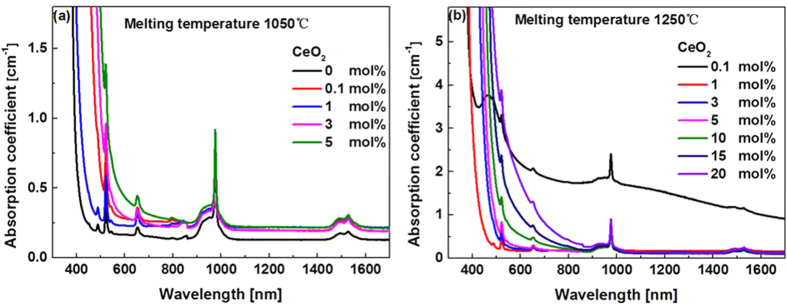
Absorption spectra of the samples marked by the rectangles in Fig. 1. (**a**) at low MT 1050 °C; and (**b**) at high MT 1250 °C.

Absorption spectra of all the studied glasses were measured (cf. Fig. S3). Here, we show only spectra of the samples enclosed by two rectangles in Fig. 1. At low MT (e.g., 1050 °C), the addition of CeO_2_ increases the absorption in the visible region such that the cut-off edge of the glasses undergoes a red-shift to longer wavelength (Fig. 2(a)). At high MT (e.g., 1250 °C), the transparency of the glasses improves markedly upon the addition of CeO_2_, while the cut-off edge again shifts to longer wavelength with CeO_2_ (Fig. 2(b)).

**Figure 3:**
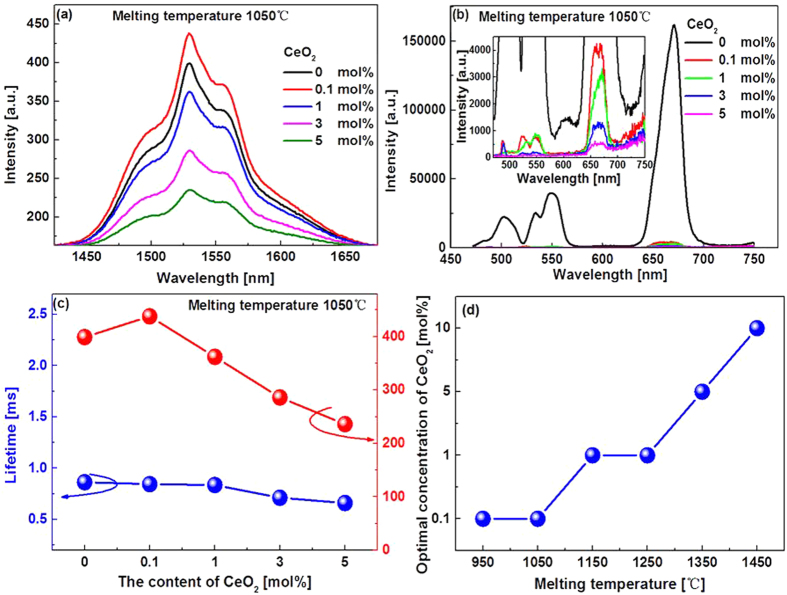
NIR (**a**), ETU (**b**) emission spectra and measured lifetime (**c**) of samples melted at the same MT (1050 °C) but doped with different concentration of CeO_2_. (**d**) Optimal doping concentration of CeO_2_ leading to the largest enhancement of the NIR emission versus MT.

The NIR emission spectra of all the studied glasses were measured (cf. Fig. S4), in all cases a very broad emission band with the full width at half maximum (FWHM) about 80 nm (Table 1), covering *S* (1460~1530 nm), *C* (1530~1565 nm) and *L* (1565~1625 nm) silica fiber communication bands, can be clearly observed. Codoping of CeO_2_ resulted in the desired variations of the emission spectra. On one hand, both the intensity and bandwidth of the emission increase upon the finite codoping of CeO_2_, for example, no more than 1 mol.% for the glasses melted at MT lower than 1050 °C. Further increase in CeO_2_, however, exerts an adverse effect on the NIR emission intensity (Fig. 3(a)). We would like to stress that even though the enhancement of the NIR emission shown in Fig. 3(a) is not that breathtaking, the result is quite reliable and reproducible, because we have tested different batches of the glasses with the same designed composition and melted under approximately the same condition, the similar enhancement was observed in every run. The CeO_2_ induced enhancement is more evident at high MT, for example, a two-fold increase in the NIR emission intensity was observed for the glasses melted at 1150 °C (cf. Fig. S4(c)). A thorough study also show that the optimal doping concentration of CeO_2_ leading to the largest enhancement is MT dependent, and increases with MT (Fig. 3(d)). The variation of the NIR emission lifetime with the content of CeO_2_ is shown in Fig. 3(c), it decreases with CeO_2_ very mildly.

On the other hand, the ETU emission almost disappears completely upon even slight co-doping of CeO_2_, and decreases gradually with increasing content of CeO_2_ (insert in Fig. 3(b)). It is noted that the red 660 nm ETU emission is unusually stronger than that at the green 532 nm wavelength which will be discussed later on.

**Figure 4:**
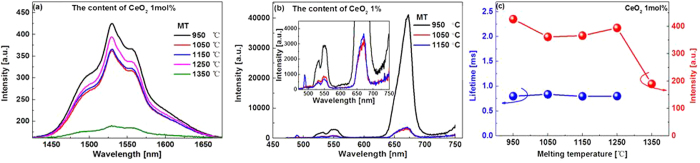
NIR (**a**), ETU (**b**) emission spectra and measured lifetime (**c**) of samples doped with the same concentration CeO_2_ but melted at different MT.

We also studied the MT dependence of the NIR and ETU emission spectra of the glasses. Fixing the doping concentration of CeO_2_ at 1 mol.%, both the NIR and ETU emissions decrease with increasing of MT (Fig. 4(a,b)), similar to our previous work^22^. However, the full width at half maximum (FWHM) of the NIR emission band remains to be 80 nm (Table 1). On the other hand, the lifetime of the NIR emission varies arbitrarily with MT (Fig. 4(c)).

**Figure 5:**
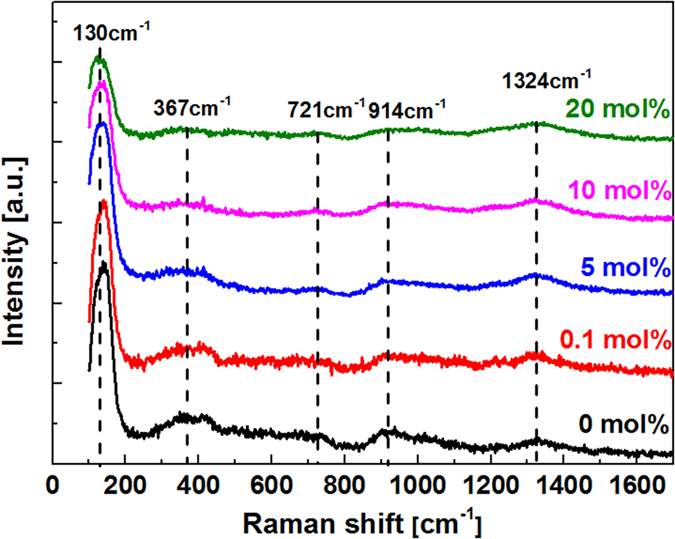
Raman spectra of samples doped with different concentration of CeO_2_.

Since the NIR emission is subject to phonon energy of glasses too, the distribution of phonon energies (lattice vibrations) of the studied glasses was examined by Raman scattering experiment, and the results are shown in Fig. 5. There are five major peaks located at ~140, 367, 721, 914, 1324 cm^−1^, respectively. The strongest peak (130 cm^−1^) attributes to the vibrational mode of Bi^3+^ in [BiO_6_] octahedron. The 367 cm^−1^ peak is caused by the distorted vibration of Bi-O-Bi and Si-O-Si in [BiO_6_] and [SiO_4_]^23^. The 721 cm^−1^ band is due to the distorted vibration of B-O-B in [BO_3_] and [BO_4_]^24^. The vibration of Bi-O-Si accounts for the peak located at 914 cm^−1^. The 1324 cm^−1^ peak is induced by the asymmetric telescopic vibration of B-O^−^ in [BO_3_] triangle and [BO_4_] tetrahedron^24^. Apparently, the network of BBS glasses is mixed with different glass forming units. With the substitution of CeO_2_ for Bi_2_O_3_, the low phonon energy modes related to [BiO_6_] decreases, while the maximum phonon energy of the glasses is invariable.

## Discussion

In the present work, the effects of CeO_2_ are two-fold. Firstly, it works as an effective oxidant to against the darkening/crystallization of the BBS glasses due to the precipitation of Bi metal or clusters during glass melting^22^. The above processes can be expressed by the following equations:


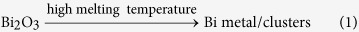



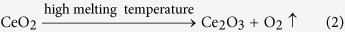



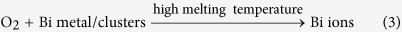


**Figure 6:**
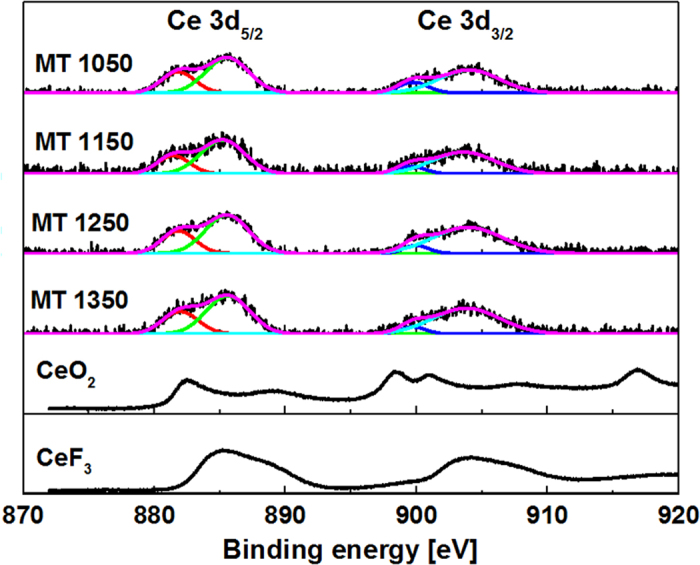
XPS of Ce 3d spin orbit doublet of samples doped with 5 mol.% of CeO_2_ but melted at different MT. Also included are the standard references CeO_2_ and CeF_3_ crystals. The XPS peaks were fitted to Gaussian function provided by Origin software.

It is the release of oxygen gas (Eq. 2) at high MT that oxidizes the Bi metal or clusters (Eq. 3) and improves the transparency of the BBS glasses (Fig. 1)^25^. According to Eq. 2, CeO_2_ itself goes into Ce_2_O_3_ during the melting, the consumption of oxygen gas by the Bi metal or clusters favors the formation of Ce^3+^ ions, especially at high MT. The XPS measurement clearly backs up this assumption (Fig. 6). Although both Ce^3+^ and Ce^4+^ can co-exist in glasses, the XPS spectra show that trivalent Ce^3+^ dominates in the studied BBS glasses, as confirmed by the resemblance of the XPS spectra to that of the standard reference CeF_3_ crystal and the absence of the characteristic doublet related to Ce^4+^ at ~916 eV^26^,^27^. However, either the presence of Ce^3+^ or Ce^4+^ can increase the absorption in the visible region up to 500 nm (Fig. 2), which is induced by either the 4*f*-5*d* transitions of Ce^3+^ or the charge transfer between O^2−^ and Ce^4+^.

**Figure 7:**
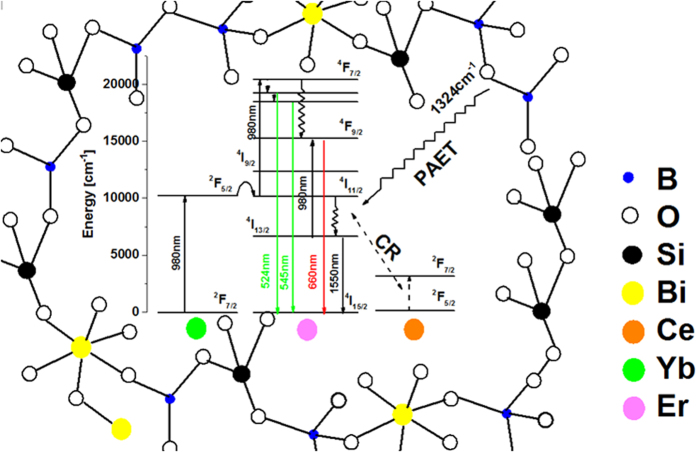
Energy diagram of Yb^3+^, Er^3+^ and Ce^3+^, and possible ET processes between these ions. The solid arrows represent radiative transitions and the curly ones non-radiative transitions (multi-phonon relaxations).

Secondly, the role of codoping CeO_2_ is to improve the NIR luminescent properties of Er^3+^. The variation of the NIR emission with the content of Ce^3+^ is complex; however it can be analyzed based on the phonon assisted energy transfer (PAET) between the relevant electronic energy levels of Er^3+^ and Ce^3+^ ions. The PAET may involve one, two or more than two phonons depending on the phonon energy of the host, concentration of and critical radii between rare earth ions (REs) in solids^28^. Generally, one phonon assisted process accounts for 20~30%, while two and more than two phonons assisted processes only 10^−6^~10^−3^ of the total transition probability of REs. Referring to the energy level diagrams of Er^3+^ and Ce^3+^ (Fig. 7), it is seen that the following two PAET processes which may influence the NIR emission: (a) Er^3+^: ^4^I_11/2_ + Ce^3+^ : ^2^F_5/2_ → Er^3+^: ^4^I_13/2_ + Ce^3+^: ^2^F_7/2_, and (b) Er^3+^: ^4^I_13/2_ + Ce^3+^: ^2^F_5/2_ → Er^3+^: ^4^I_15/2_ + Ce^3+^ :^2^F_7/2_ have energy mismatches approximately 1400 cm^−1^ and 4300 cm^−1^, respectively. The maximum phonon energy of the glasses is 1350 cm^−1^ (Fig. 5), which means that the former process (a) leading to the increased population of the NIR lasing level ^4^I_13/2_ of Er^3+^ and enhanced NIR emission requires one phonon, while the latter (b) more than two phonons. Although the latter may quench the NIR emission, it hardly plays a role for low doping concentration, because the PAET involving more than two phonons generally has extremely low efficiency. In fact, the luminescence decay measurement (cf. Table S2) shows that the lifetime of the NIR luminescence varies by only 5% when the content of Ce^3+^ is low (<1 mol.%).

However, at too high concentration of Ce^3+^ (e.g., >5 mol.%), one phonon assisted process may also occurs between one Er^3+^ ion and a pair of Ce^3+^ ions (via Er^3+^: ^4^I_13/2_ + Ce^3+^: ^2^F_5/2_ + Ce^3+^: ^2^F_5/2_ → Er^3+^: ^4^I_15/2_ + Ce^3+^: ^2^F_7/2_ + Ce^3+^: ^2^F_7/2_). The energy transfer efficiency (*η*) between Er^3+^ and Ce^3+^ can be calculated according to the equation: *η* = 1 − *τ*/*τ*_0_, where *τ* and *τ*_0_ are lifetimes of the donor (Er^3+^) with and without acceptor (Ce^3+^). A rough estimate of *η* shows that it equals to 30~60% for high doping concentration of Ce^3+^, suggesting that one phonon assisted process indeed occurs. A more rigorous calculation of the PAET efficiency requires knowledge of the absorption cross-section of the Ce^3+ 2^F_7/2_ → ^2^F_7/2_ transition (~2200 cm^−1^), which is impossible to obtain because the intrinsic phonon cut-off edge of the samples lies at shorter wavelength (~3000 cm^−1^). Incidentally, the PAET has also been found a very successful means to improve the lasing efficiency of Yb^3+^/Ho^3+^ and Yb^3+^/Tm^3+^ pairs in solids^29^. We have failed to measure the lifetimes of ^4^I_11/2_ level of Er^3+^ before and after codoping of CeO_2_ because the 1000 nm and 2.7 μm emissions from ^4^I_11/2_ level were too weak to be recorded, thus we are not able to deduce the PAET efficiency between Er^3+^ and Ce^3+^.

One may have noticed that the lifetime of the 1.55 μm emission of Er^3+^ was rather short (~1 ms) in the studied glasses (Fig. 3(c)). This is because the glasses contain a large amount of B_2_O_3_ as high as 50 mol.%, giving rise to much faster multi-phonon relaxation between the lasing level ^4^I_13/2_ and the ground level ^4^I_15/2_ as compared to glasses containing a less amount of B_2_O_3_ (~3 ms)^30^. In this respect, it seems preferable to use glasses containing a limited amount of B_2_O_3_, however, when referring to our previous work^22^ and other published papers^30^, it appears that the broadband NIR emission with a FWHM over 80 nm can only be obtained in glasses containing relatively high content of B_2_O_3_^8^. Since the emission bandwidth of rare-earth (RE) ions is largely determined by the variations of ligand fields around RE ions from site to site (inhomogeneous broadening), and the ligand fields around RE ions are mainly influenced by glass formers, consequently, the broadband NIR emission observed in the BBS glasses can be partially accounted for by the so-called “mixed glass former effect” due to the presence of a multiplicity of glass former motifs (Fig. 5)^31^. However, a compromise indeed exists between the emission lifetime and the bandwidth regarding the optimized concentration of B_2_O_3_. Fortunately, because Bi_2_O_3_-B_2_O_3_-SiO_2_ ternary system has a very broad glass forming region, providing a rich tuning range of glass compositions, the lasing and other relevant physical/ optical properties are amenable to being tailored for specific requirements. Work is now under way to further refine the lasing properties of the glasses and to understand the line broadening mechanism induced by B_2_O_3_.

Previous report showed that when the content of B_2_O_3_ increased to 8 mol.% in Er^3+^/Yb^3+^ codoped tungsten-tellurite glasses, the red ETU emission was already getting stronger than the green one^32^. This phenomenon is also owing to the presence of HPE compound B_2_O_3_, which tends to decrease the lifetime of ^4^I_11/2_ level and as such increase the population of ^4^I_13/2_ level, consequently, the ETU emission involving the intermediate ^4^I_11/2_ level, viz., the green one, was reduced, whereas the red one involving the intermediate ^4^I_13/2_ level enhanced. As mentioned above, the studied glasses contain a high concentration of B_2_O_3_, it is no wonder that the red ETU is stronger than that of the green one (Fig. 3(b)).

Judd Ofelt (JO) analysis is the most useful tool in estimating the forced electric dipole transitions of RE ions. JO parameters can be derived from the absorption spectra and the results are shown in Table 1. According to the theory, three JO intensity parameters (Ω_t_, t = 2, 4, 6), which depend on the local chemical environments, determine the radiative transition rate of RE ions. Of the three parameters, Ω_2_ is the most sensitive to local structure, and previous studies have suggested that it increases with the degree of asymmetry around RE ions^33^,^34^. The other two parameters, Ω_4_ and Ω_6_ are not so much susceptible to the environmental changes as Ω_2_. However, Ω_6_ has been found to increase with the extent of the overlap between the 4*f* and 5*d* orbitals of Er^3+^ ions, or instead, it decreases as the 5*d* electron density diminishes. The substitution of CeO_2_ for Bi_2_O_3_ reduces the number of highly polarizable Bi-O bonds (Fig. 5), and according to our previous study^22^, this may in turn decrease to some extent the microsymmetries around Er^3+^ ions, which partly accounts for the increased Ω_2_.

**Figure 8:**
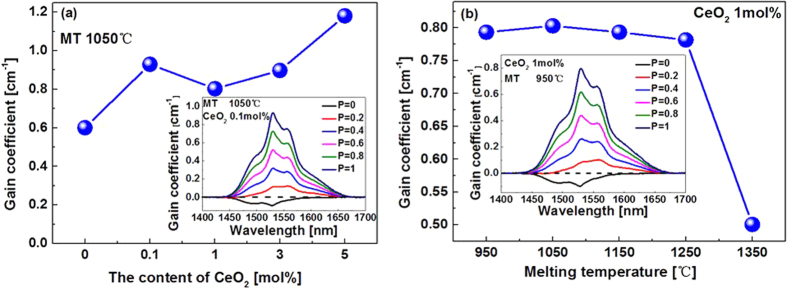
Variation of gain coefficient of samples as a function of CeO_2_ (**a**) and MT (**b**). Insets: typical gain spectra for demonstration.

Gain coefficient of the NIR emission, *G*(*λ*), was calculated according to 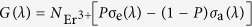, where *P* is the population inversion given by the ratio between the population of Er^3+^ ions at the lasing level (^4^I_13/2_) and total concentration (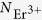) of Er^3+^ ions, *σ*_a_(λ) is the absorption cross-section, *σ*_e_(λ) is the emission cross-section which can be calculated according to Fuchtbauer-Ladenburg equation:
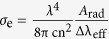
, where *c* is the speed of light, *n* is the refractive index and *A*_rad_ is the radiative transition rate obtained from JO analysis, Δ*λ*_eff_ was calculated by 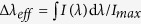. Positive gain already appears when only twenty percent of Er^3+^ ions are populated to the lasing level (insert in Fig. 8(a)). The maximum gain increases with the concentration of CeO_2_. It should be mentioned that the gain may also depend on the ESA of Er^3+^ as in the case of Er^3+^-doped silica fibers^35^,^36^, so in reality, the calculated *G*(*λ*)-values can suffer from overestimating. On the other hand, with the fixed doping concentration of CeO_2_, the gain coefficient barely changes with MT (Fig. 8(b)).The sudden drop in the gain value is due to the crystallization of the samples melted at high MT.

## Conclusion

Depending on the melting temperature, a large amount of CeO_2_ (up to 20 mol.%) can be doped into the bismuth borosilicate (BBS) glasses, and cerium ions present as trivalent oxidation state Ce^3+^ overwhelmingly. The benefits of the doping of CeO_2_ include, on one hand, improving significantly the transparency of the glasses especially for those melted at high temperatures; on the other hand, enhancing the 1.55 μm near-infrared (NIR) emission without sacrificing the lifetime due to the phonon-assisted energy transfer (PAET) between Er^3+^ and Ce^3+^. However, the doping concentration must be limited because otherwise both the intensity and lifetime of the NIR emission degrade. In addition, the undesirable energy transfer up-conversion (ETU) emission and excited state absorption of Er^3+^ are severely quenched by doping of CeO_2_ stemmed from the accelerated non-radiative transition ^4^I_11/2_ → ^4^I_13/2_ of Er^3+^. The unique combination of enhanced NIR emission intensity, broadened NIR emission bandwidth and suppressed ETU emission makes Ce^3+^/Yb^3+^/Er^3+^ triply doped BBS glass a potential fiber material for broadband EDFAs. And because Bi_2_O_3_-B_2_O_3_-SiO_2_ system has a very broad glass forming region, providing a rich compositional tuning range, the lasing and other related physical/optical properties can be tailored for specific requirements. The strategy described here, namely, de-excitation by PAET between rare-earth (RE) ions, increasing non-radiative transition by using high phonon energy compounds and line broadening through mixed glass former effect, can be readily extended to other RE pairs such as Yb^3+^-Tm^3+^ and Yb^3+^-Ho^3+^ to enhance the 2.0 μm emissions of Tm^3+^ and Ho^3+^ doped solids. More work has to be done on optimizing the concentration of B_2_O_3_ and exploiting the physical basis for the line broadening induced by B_2_O_3_.

## Additional Information

**How to cite this article**: Chu, Y. *et al*. Ce^3+^/Yb^3+^/Er^3+^ triply doped bismuth borosilicate glass: a potential fiber material for broadband near-infrared fiber amplifiers. *Sci. Rep.* **6**, 33865; doi: 10.1038/srep33865 (2016).

## Supplementary Material

Supplementary Information

## Figures and Tables

**Table 1:** JO parameters Ω_t_ (t = 2, 4, 6, ×10^−2^ cm^−2^)*, FWHM (nm), σ_e_ (×10^−2^ cm^2^), τ (ms), σ_e_ × FWHM (×10^−^ nm^3^) and σ_e_ × τ (×10^−2^ cm^2^·s) of the samples.

Glasses		Ω_2_	Ω_4_	Ω_6_	FWHM	σ_e_	τ	σ_e_ × FWHM	σ_e_ × τ
MT (°C)	CeO_2_ (mol%)								
1050	0	3.4	2.34	0.96	75.17	0.70	0.86	52.62	6.04
	0.1	5.61	3.6	1.56	78.67	1.02	0.84	80.24	8.61
	1	5.15	3.37	1.36	79.87	0.99	0.82	79.07	8.28
	3	4.93	3.41	1.51	77.13	1.41	0.71	108.75	10.01
	5	7.19	3.48	2.01	79.28	2.54	0.66	201.37	16.76
950	1	5.74	3.68	1.58	80.75	1.03	0.80	83.17	8.24
1050		5.15	3.37	1.36	79.87	0.99	0.82	79.07	8.31
1150		5.45	3.69	1.33	80.75	0.98	0.80	79.14	7.84
1250		5.28	3.32	1.34	79.96	0.93	0.80	74.36	7.44

*The error of fitting is no more than 15% for all the samples.
