# Supplementary material for: Ce3+/Yb3+/Er3+ triply doped bismuth borosilicate glass: a potential fiber material for broadband near-infrared fiber amplifiers
Source: Sci Rep. 2016 Sep 20;6:33865. doi: 10.1038/srep33865 (PMC5028740; doi:10.1038/srep33865)
Supplement: Supplementary Information [file srep33865-s1.docx]

Supplementary materials for

**Ce^3+^/Yb^3+^/Er^3+^ triply doped bismuth borosilicate glass: a potential fiber material for broadband near-infrared fiber amplifiers**

**Yushi Chu ^1^, Jing Ren ^1,*^, Jianzhong Zhang ^1,*^, Gangding Peng ^2^, Jun Yang ^1^,**

**Pengfei Wang ^1^ and Libo Yuan ^1^**

^1^Key Lab of In-fiber Integrated Optics, Ministry Education of China, Harbin Engineering University, Harbin 150001, China

^2^Photonics & Optical Communications, School of Electrical Engineering & Telecommunications, University of New South Wales, Sydney 2052, NSW, Australia

Corresponding author: ren.jing@hrbeu.edu.cn (J. Ren), zhangjianzhong@hrbeu.edu.cn (J. Zhang)

The XRD patterns of the samples sitting on the border (Fig. S1(a)) separating those that can form glass and those cannot were measured by Rigaku D/max 2500 with the scanning speed 8 °/min., and the results are shown in Fig. S1(b). The amorphous nature of these glasses was confirmed. However, the XRD patterns of those samples with the compositions and MT as indicated by the empty space in Fig. S1(a) were not measured because the crystallization can be inferred from the fact that the samples became totally opaque and serious ceramization occurred which can be observed even by the naked eyes.


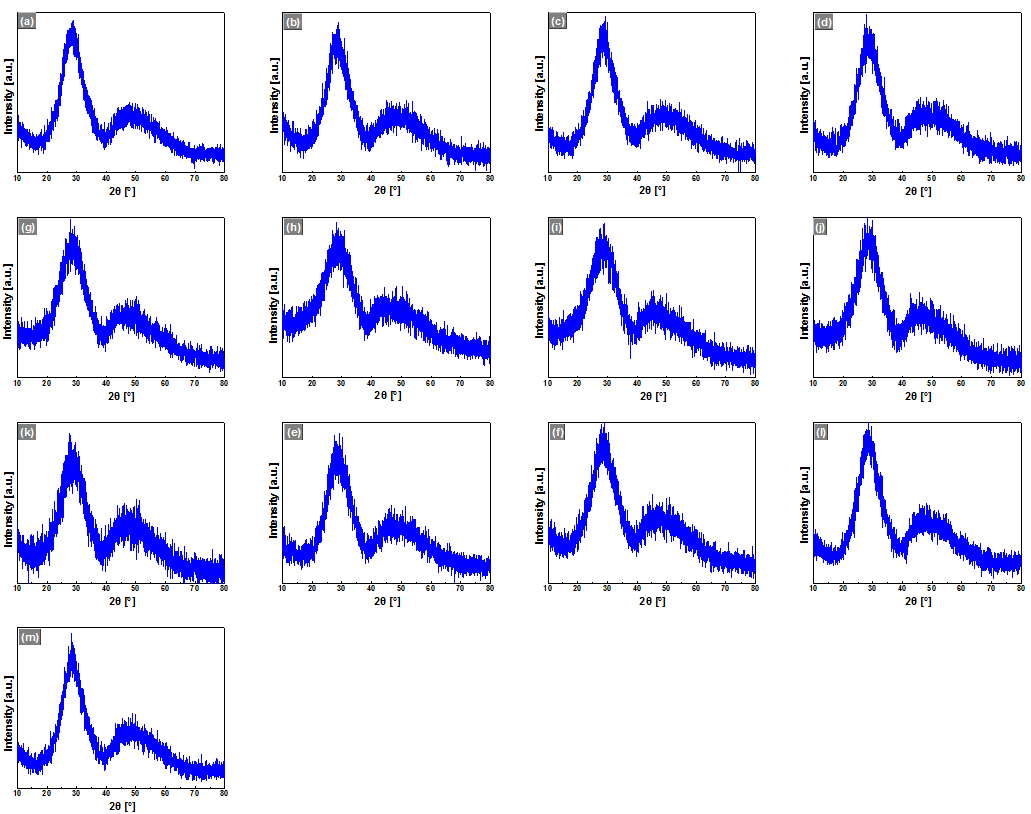


Fig. S1 (a) The samples chosen for XRD measurement. (b) XRD patterns of the samples sitting on the border separating those that can form glass and those cannot.

Thermal analysis experiments were performed using a differential scanning calorimeter (DSC, TA D2000, USA) from room temperature to 900 °C at a heating rate of 10 °C/min, and samples were firstly crushed into fine powder. Figure S2 shows the DSC curves of some representative samples. The characteristic temperatures of the glasses (T_g_, T_x_) are shown in Table S1. Except for the sample melted at the highest MT, all the other samples did not show any obvious crystallization peak and the ΔT = T_x_-T_g_ (T_x_, onset temperature of crystallization and T_g_, glass transition temperature) values were considerably larger than 100 °C, indicating excellent thermal stability and fiber drawing potential of the glasses.

| Samples | *T*_g_/ ^o^C | *T*_x_/ ^o^C | Δ*T*/ ^o^C |
| --- | --- | --- | --- |
| a | 466 | 643 | 177 |
| b | 492 | 673 | 181 |
| c | 474 | 645 | 171 |
| d | 638 | 718 | 80 |


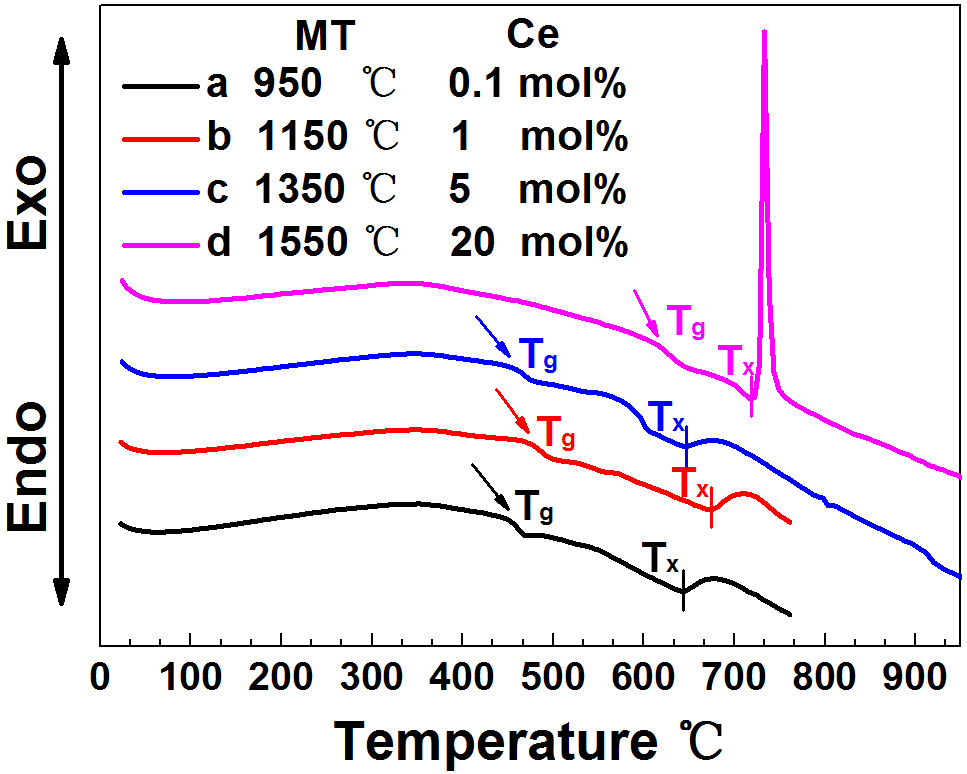


Table S1 Glass transition temperature (T_g_), onset crystallization temperature (T_x_) and their difference (ΔT) determined from DSC curve

Fig. S2 DSC curves for some representative samples that show the best NIR luminescent properties according to Fig. 3(d) of the revised paper

Absorption spectra of all the studied glasses were measured. Here, S3(a)~(g) are the absorption spectra of the samples doped with different concentration of CeO_2_ but melted at fixed temperatures ranging from 950 ^o^C to 1550 ^o^C with an interval of 100 ^o^C. On the other hand, S3(h)~(o) represent the absorption spectra of the glasses melted at different temperatures ranging from 950 ^o^C to 1550 ^o^C with an interval of 100 ^o^C but doped with fixed concentration of CeO_2_.


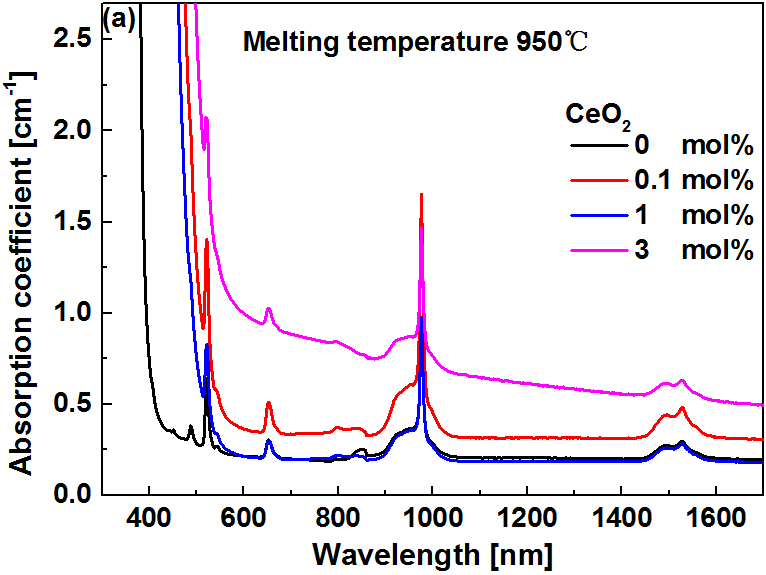

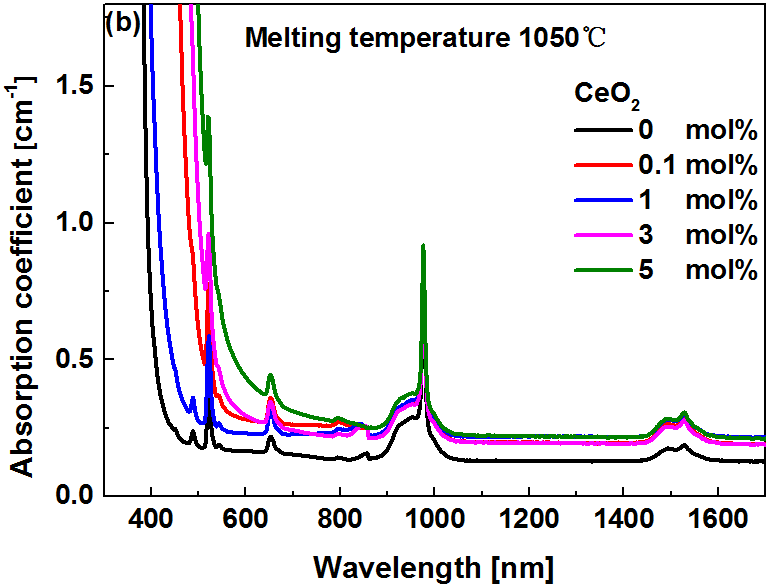


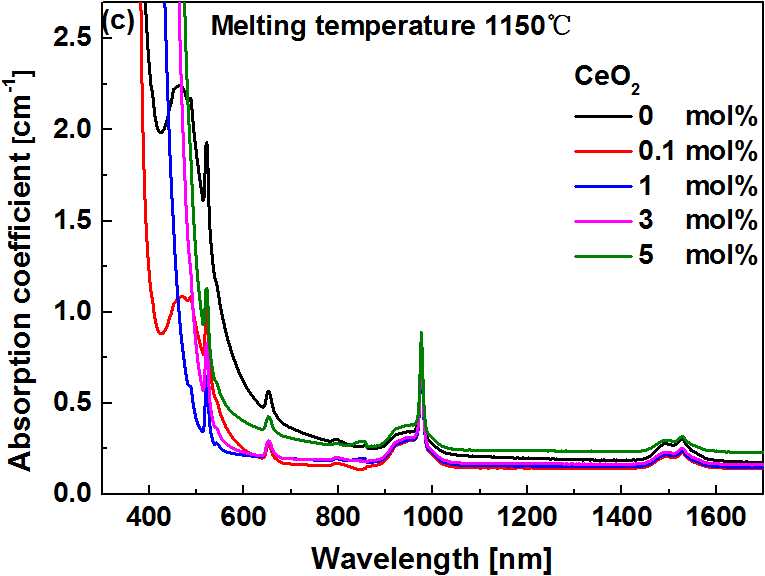

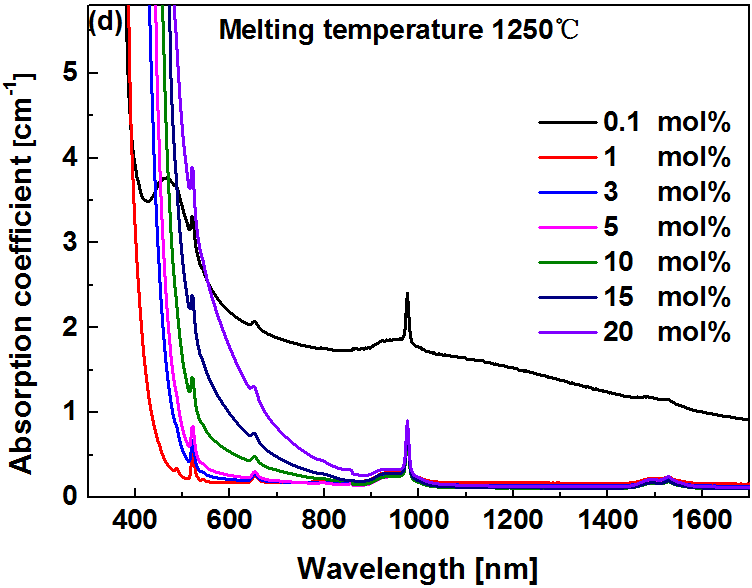


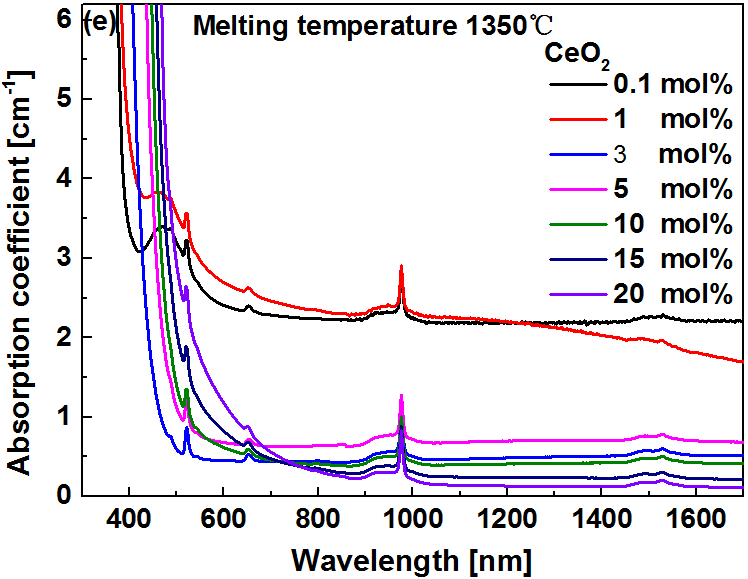

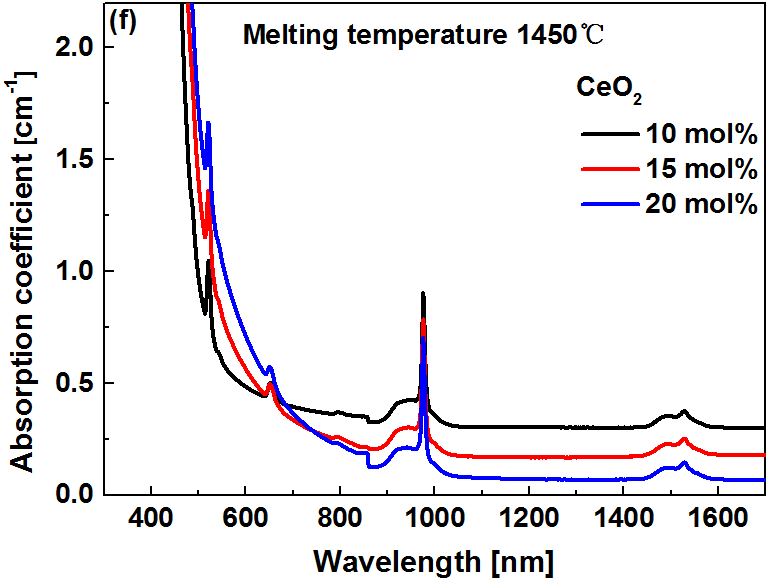


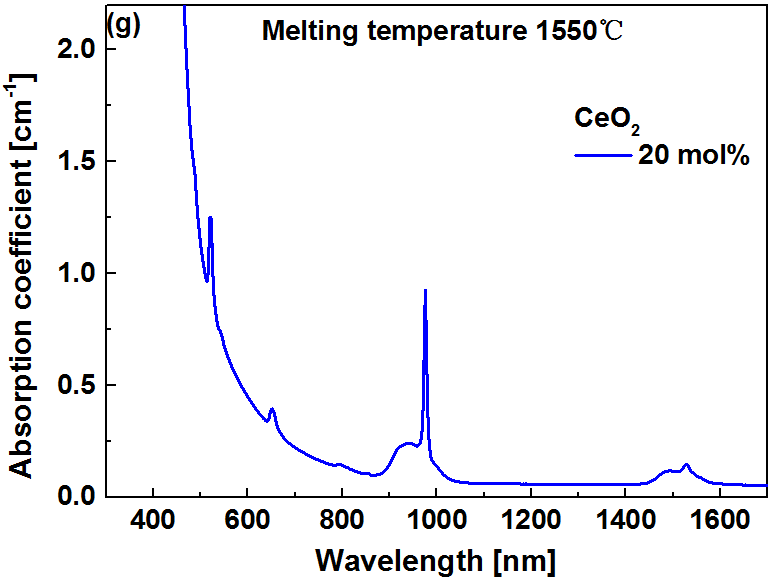


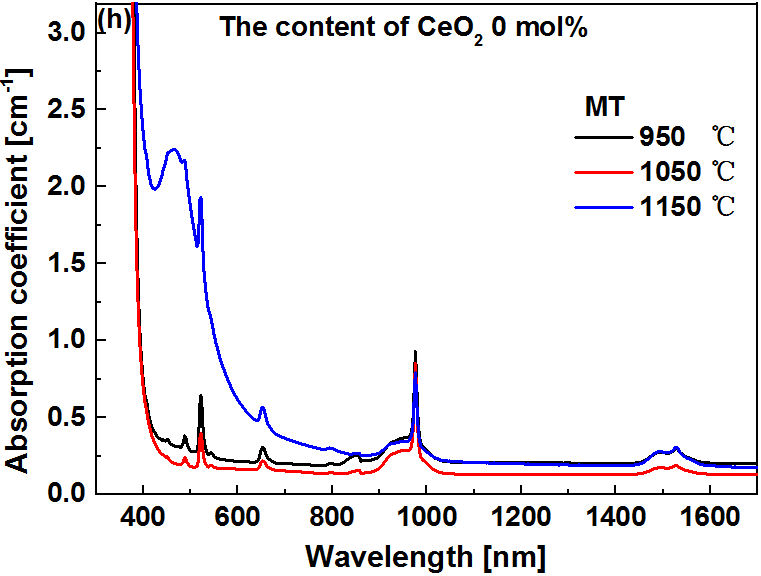

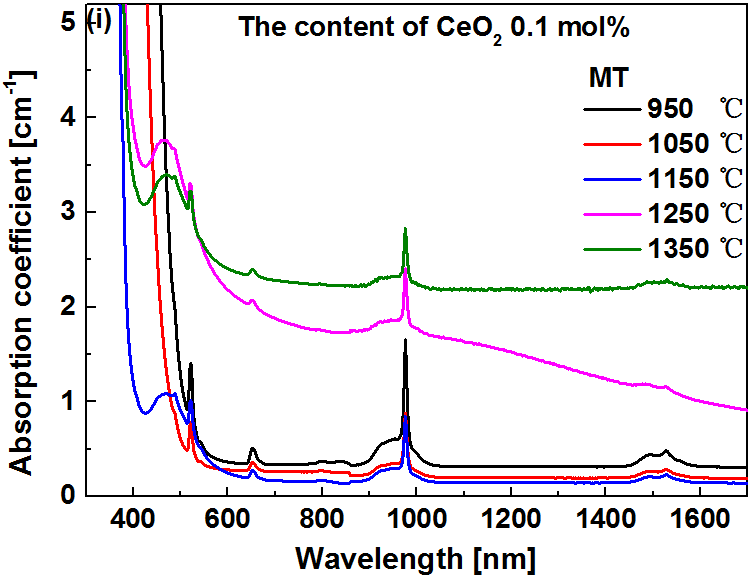

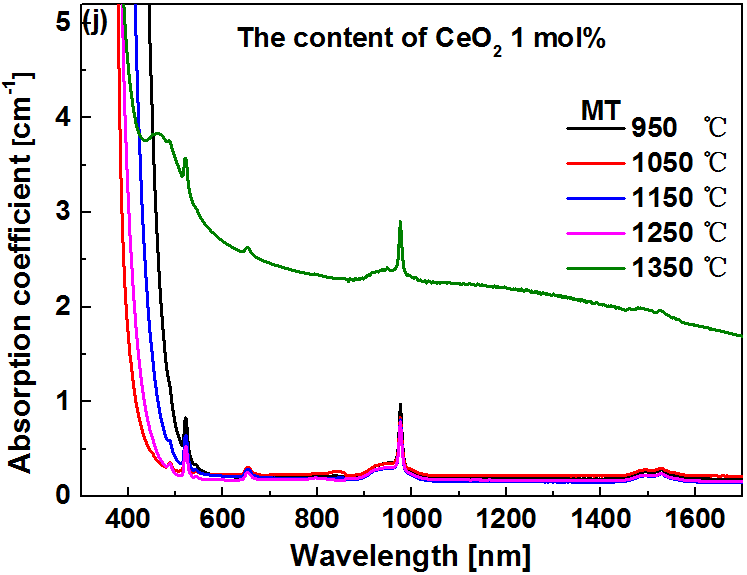

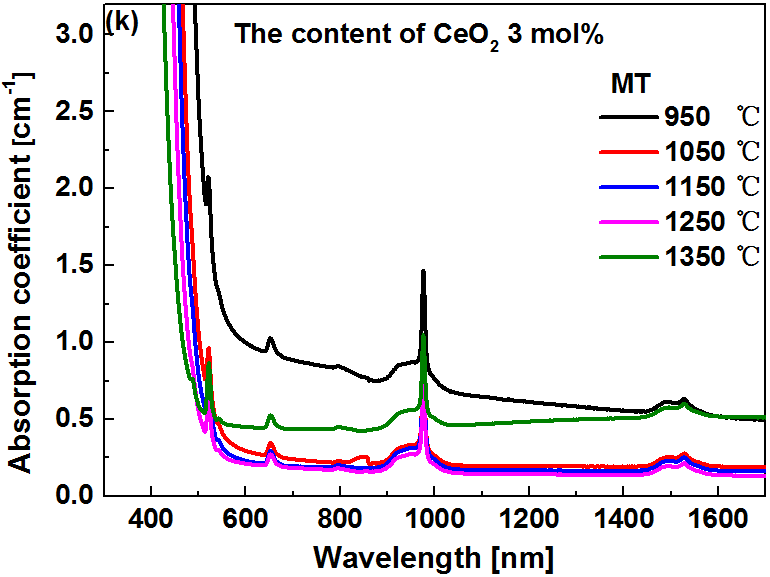

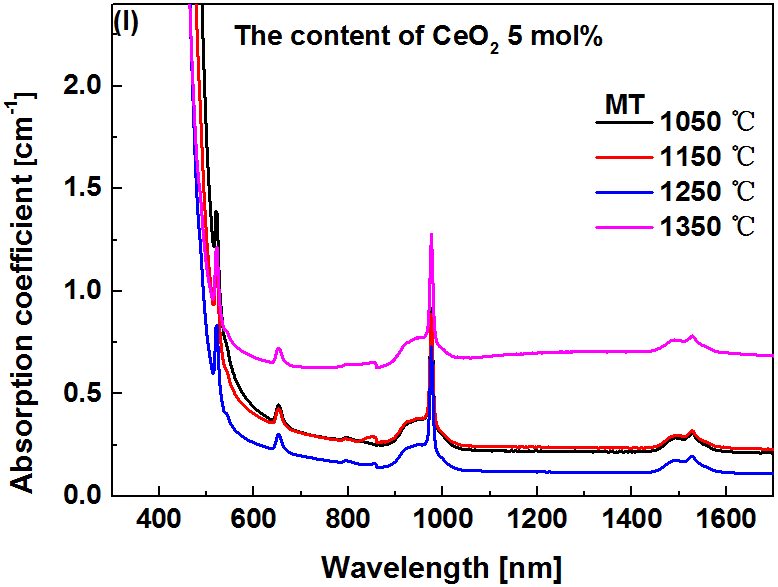

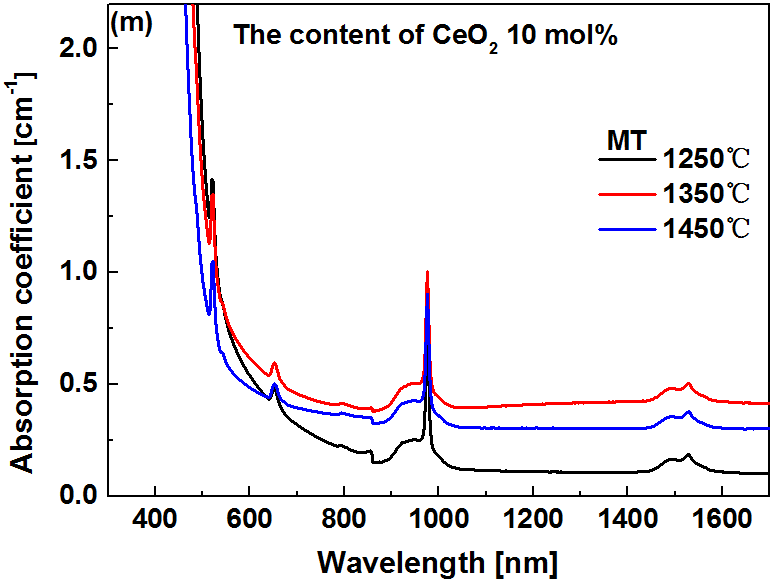

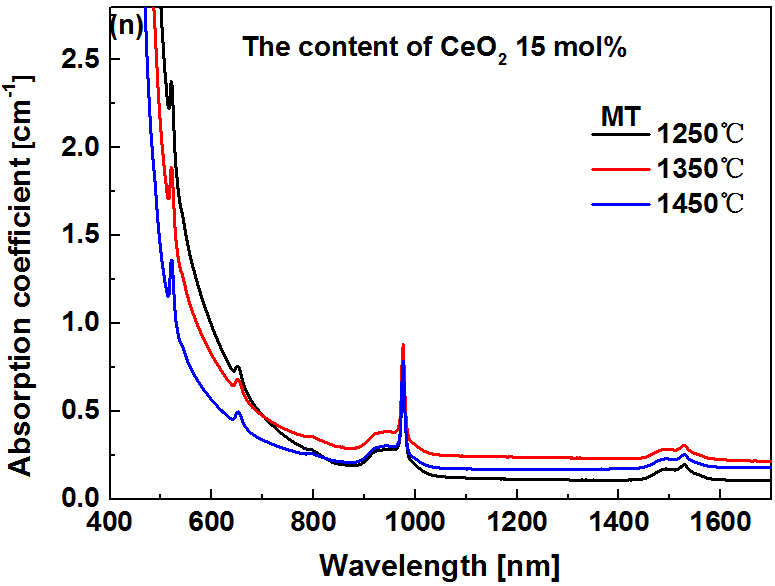

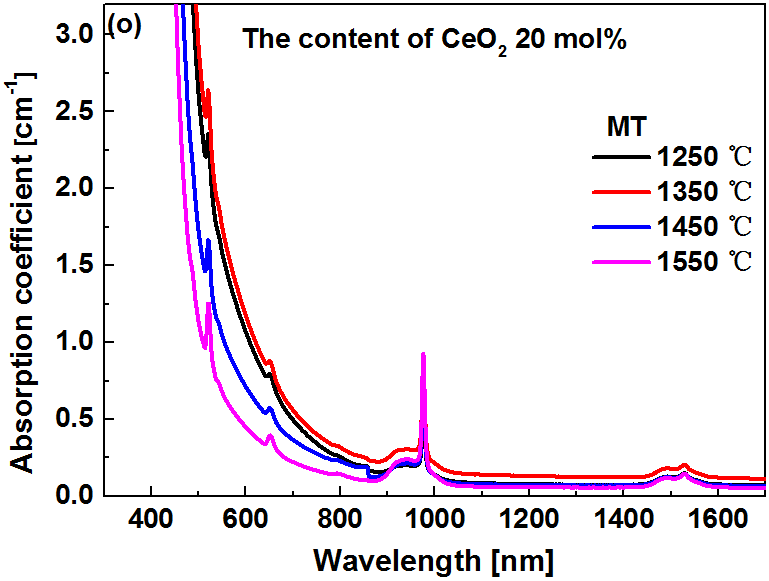


Fig. S3 Absorption spectra of the samples melted at the same temperature but doped with different concentration of CeO_2_ (a)~(g) and doped with the same concentration of CeO_2_ but melt at different temperature (h)~(o).

NIR spectra of all the studied glasses were measured. Here, S4(a)~(g) are the NIR spectra of the samples doped with different concentration of CeO_2_ but melted at fixed temperatures ranging from 950 ^o^C to 1550 ^o^C with an interval of 100 ^o^C. On the other hand, S4(h)~(o) represent the NIR spectra of the glasses melted at different temperatures ranging from 950 ^o^C to 1550 ^o^C with an interval of 100 ^o^C but doped with fixed concentration of CeO_2_. Table S2 shows the lifetimes of the samples.


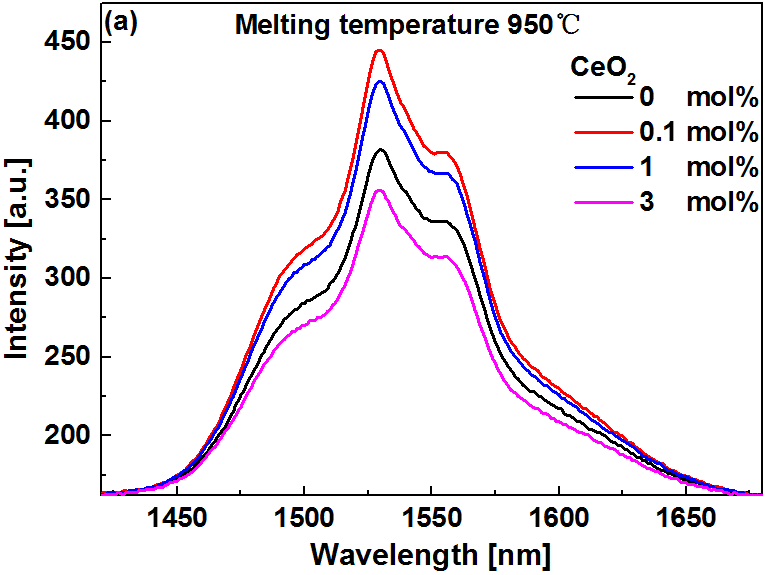

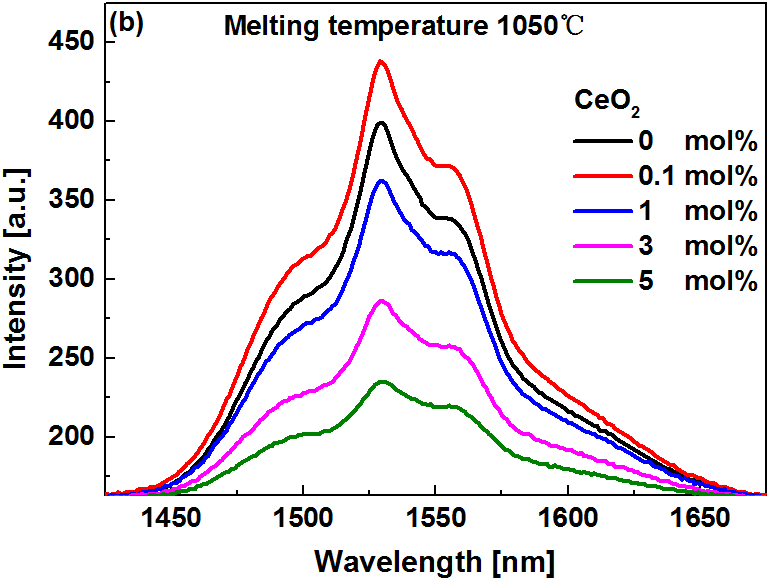


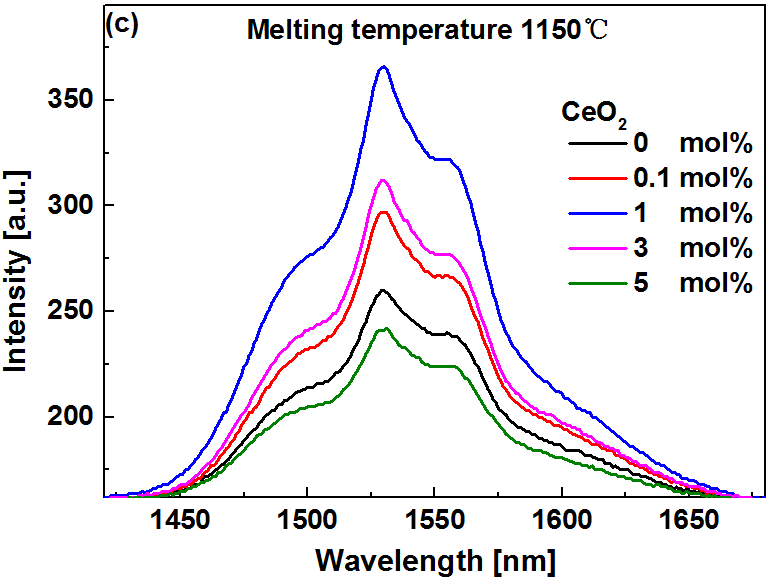

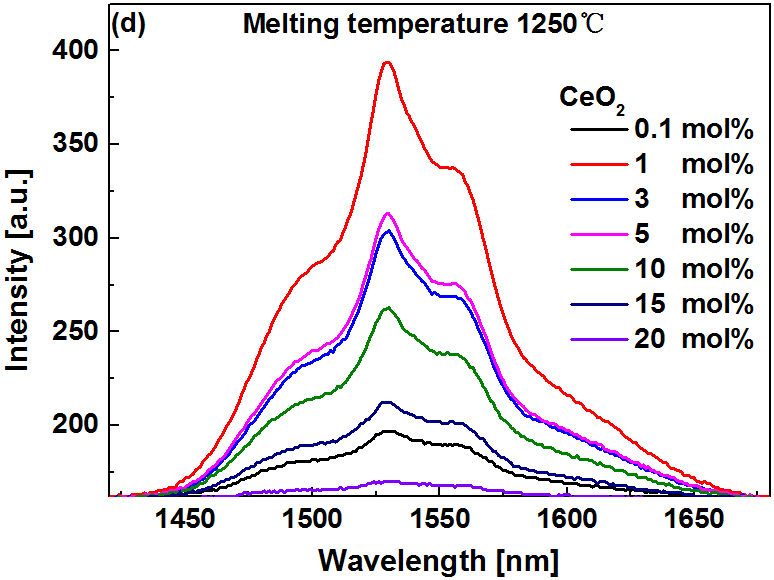


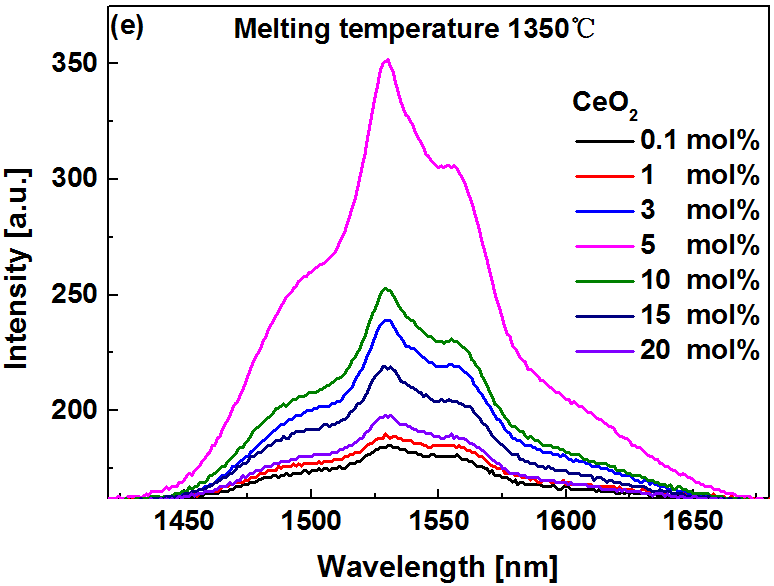

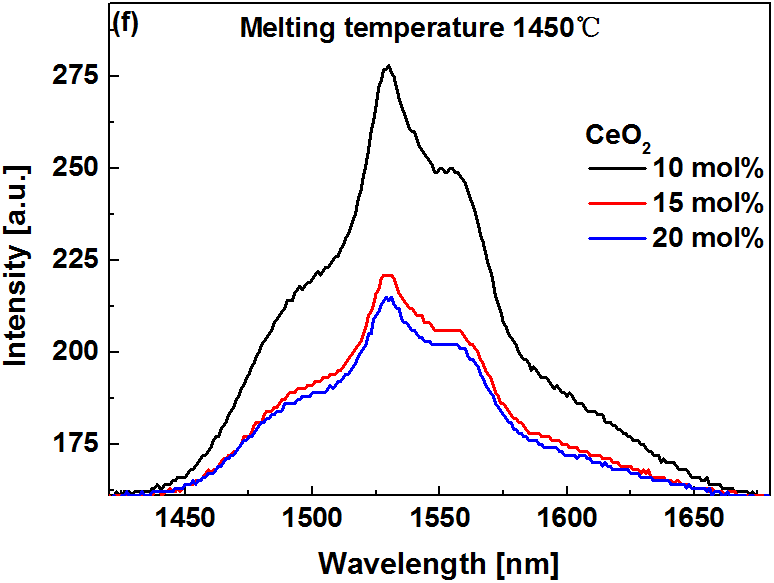


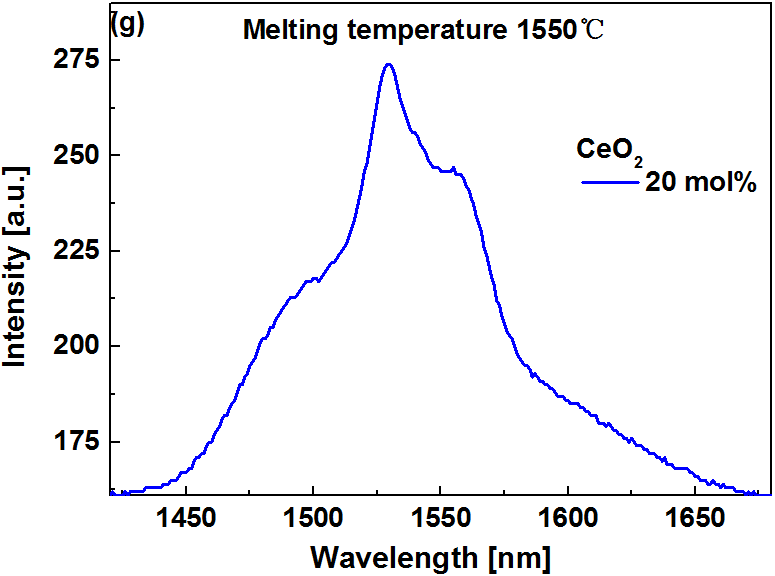


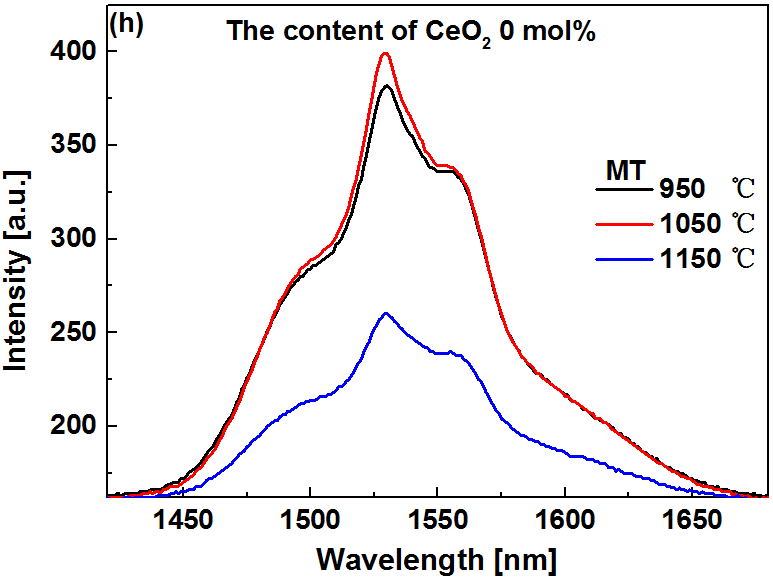

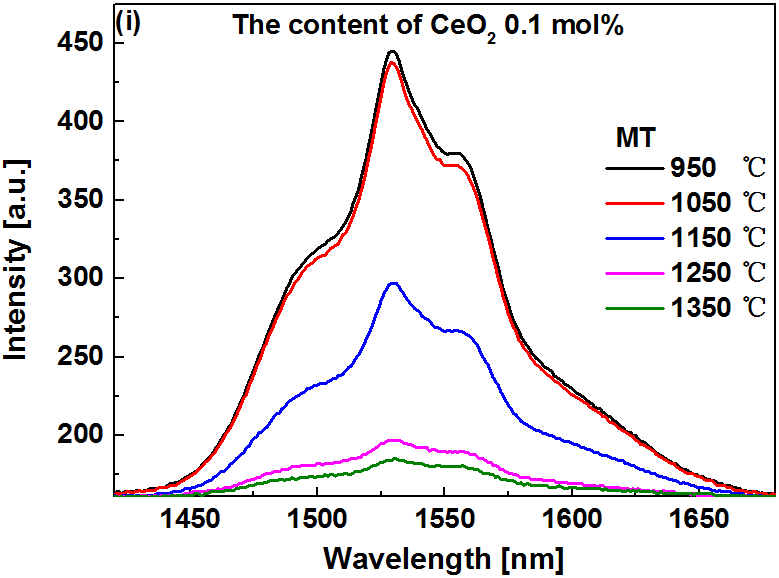


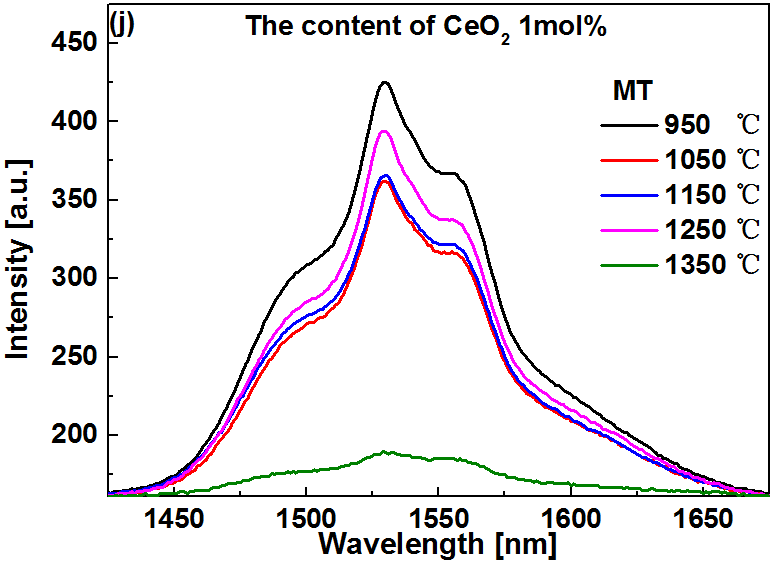

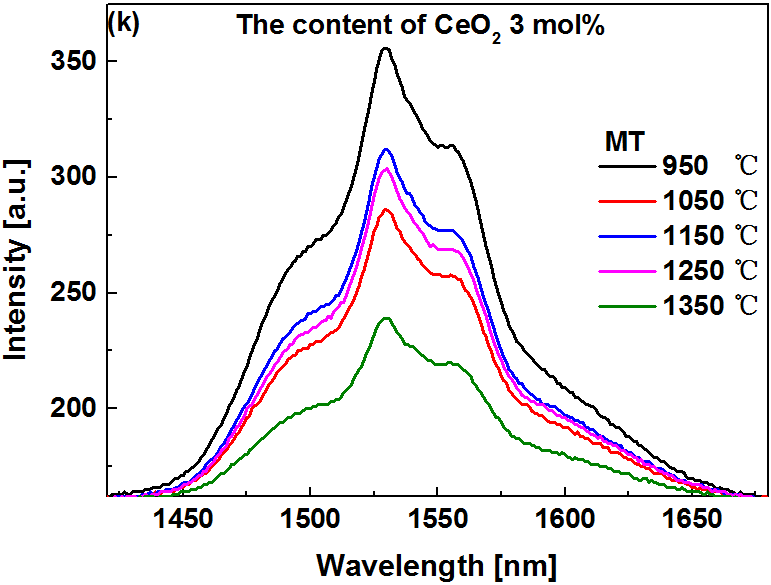


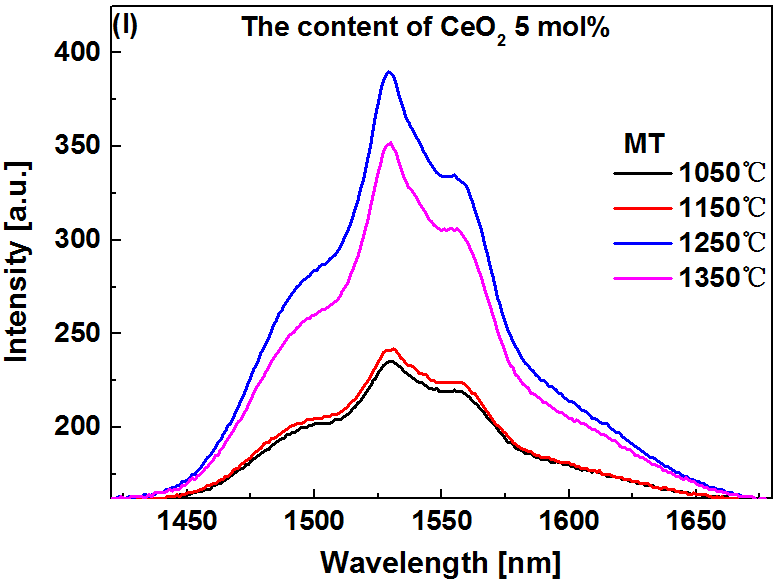

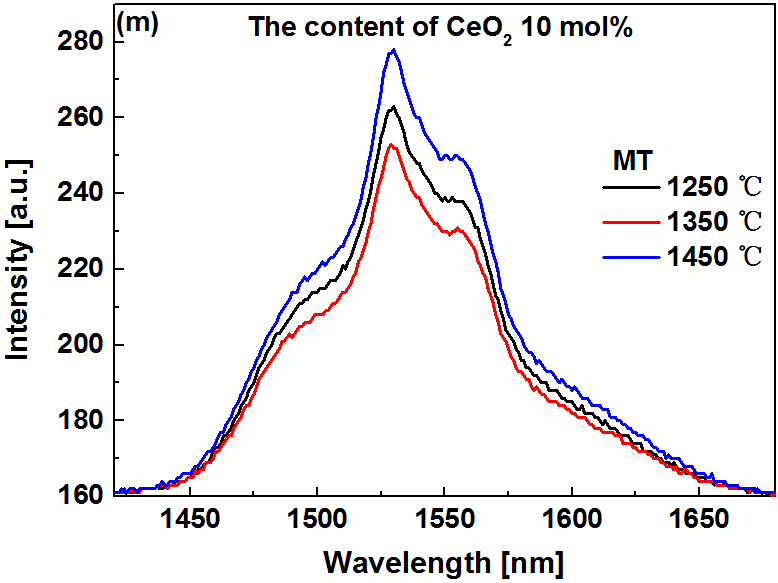


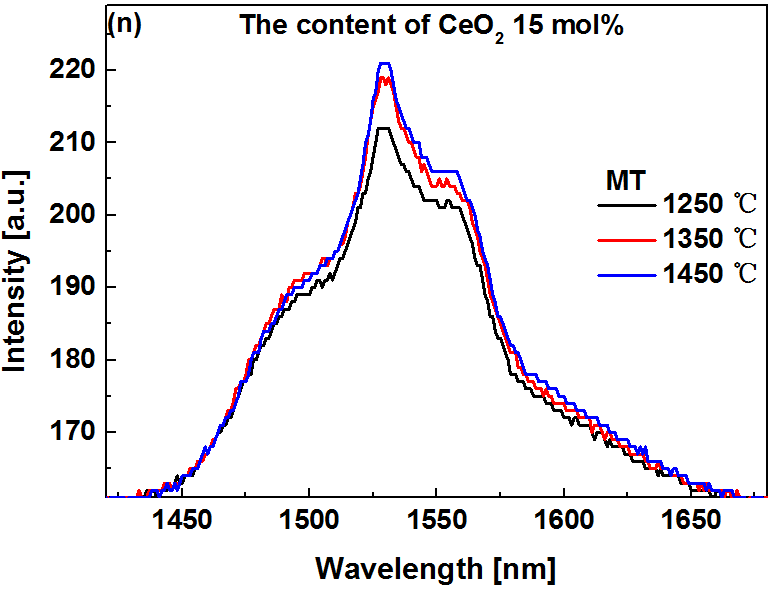

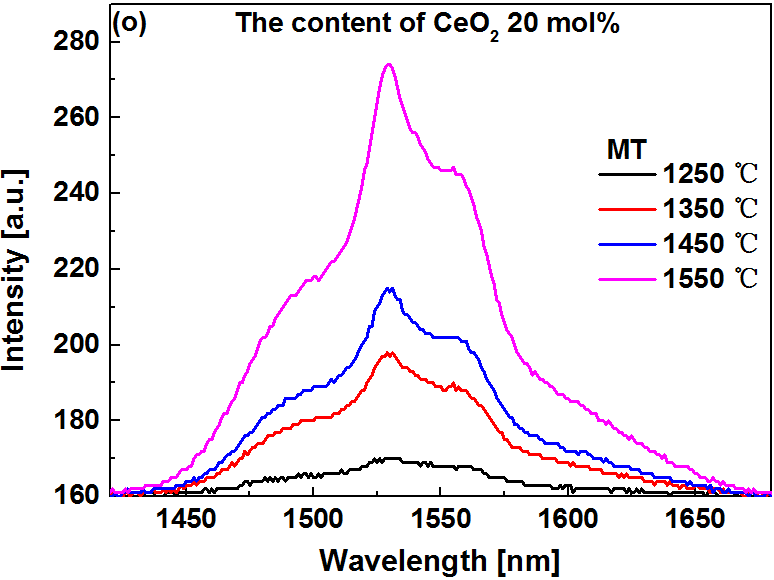


Fig. S4 NIR spectra of the samples melted at the same temperature but doped with different concentration of CeO_2_ (a)~(g) and doped with the same concentration of CeO_2_ but melt at different temperature (h)~(o).

Table S2. The lifetimes of the samples (first line: MT/℃, first column: content of CeO_2_/mol%, others: lifetime/ms).

|  | 950 | 1050 | 1150 | 1250 | 1350 | 1450 | 1550 |
| --- | --- | --- | --- | --- | --- | --- | --- |
| 0 | 0.82239 | 0.86316 | 0.71013 |  |  |  |  |
| 0.1 | 0.79628 | 0.84393 | 0.7623 |  |  |  |  |
| 1 | 0.79873 | 0.83584 | 0.79546 | 0.80001 |  |  |  |
| 3 |  | 0.71151 | 0.69277 | 0.73256 | 0.73664 |  |  |
| 5 |  | 0.65987 |  | 0.75545 | 0.81855 |  |  |
| 10 |  |  |  | 0.534 | 0.63328 | 0.68069 |  |
| 15 |  |  |  | 0.39088 | 0.5079 | 0.58548 |  |
| 20 |  |  |  | 0.3127 | 0.4229 | 0.49719 | 0.51935 |

Figure S5 are the refractive indices of the samples, (a) MT: 1050℃, the content of CeO_2_: 0~5 mol%, (b) MT: 950~1250 ℃, the content of CeO_2_: 1 mol%. Refractive index was measured by using variable angle spectroscopic ellipsometry (VASE, J. A. Woollam Co., Incellipsometry) with rotating analyzers. Cauchy model was used for the fitting..


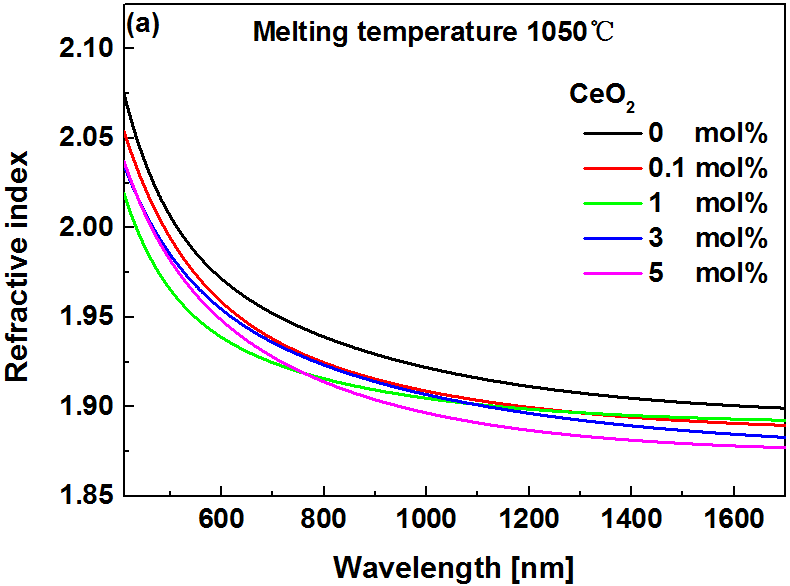

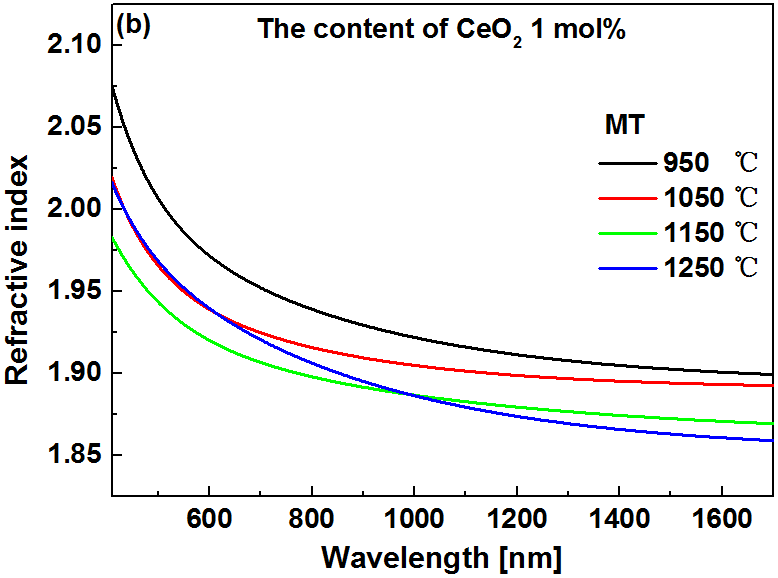


Fig. S5 Refractive indices of the samples, (a) melting temperature and the content of CeO_2_ is 1050 ^o^C and 0~5 mol%, respectively, (b) melting temperature and the content of CeO_2_ is 950~1250 ^o^C and 1mol%, respectively.
